# Timing of physical therapy consultation on 1-year healthcare utilization and costs in patients seeking care for neck pain: a retrospective cohort

**DOI:** 10.1186/s12913-018-3699-0

**Published:** 2018-11-26

**Authors:** Maggie E. Horn, Julie M. Fritz

**Affiliations:** 1 Department of Orthopaedic Surgery, Doctor of Physical Therapy Division, Duke University, Box 104002, Durham, NC 27708 USA; 2 Department of Physical Therapy, University of Utah, 520 Wakara Way, Salt Lake City, UT 84108 USA

**Keywords:** Neck pain, Healthcare utilization, Costs, Opioids

## Abstract

**Background:**

Patients seek care from physical therapists for neck pain but it is unclear what the association of the timing of physical therapy (PT) consultation is on 1-year healthcare utilization and costs. The purpose of this study was to compare the 1-year healthcare utilization and costs between three PT timing groups: patients who consulted a physical therapist (PT) for neck pain within 14 days (early PT consultation), between 15 and 90 days (delayed PT consultation) or between 91 and 364 days (late PT consultation).

**Methods:**

A retrospective cohort of 308 patients (69.2% female, ages 48.7[±14.5] years) were categorized into PT timing groups. Descriptive statistics were calculated for each group. In adjusted regression models, 1-year healthcare utilization of injections, imaging, opioids and costs were compared between groups.

**Results:**

Compared to early PT consultation, the odds of receiving an opioid prescription (aOR = 2.79, 95%CI: 1.35–5.79), spinal injection (aOR = 4.36, 95%CI:2.26–8.45), undergoing an MRI (aOR = 4.68, 95%CI:2.25–9.74), X-ray (aOR = 2.97, 95%CI:1.61–5.47) or CT scan (aOR = 3.36, 95%CI: 1.14–9.97) were increased in patients in the late PT consultation group. Similar increases in risk were found in the delayed group (except CT and Opioids). Compared to the early PT consultation group, mean costs were $2172 ($557, $3786) higher in the late PT contact group and $1063 (95%CI: $ 138 - $1988) higher in the delayed PT consultation group.

**Discussion:**

There was an association with the timing of physical therapy consultation on healthcare utilization and costs, where later consultation was associated with increases costs and healthcare utilization. This study examined the association of timing of physical therapy consultation on costs and healthcare utilization, but not the association of increased access to physical therapy consultation. Therefore, the findings warrant further investigation to explore the effects of increased access to physical therapy consultation on healthcare utilization and costs in a prospective study.

**Electronic supplementary material:**

The online version of this article (10.1186/s12913-018-3699-0) contains supplementary material, which is available to authorized users.

## Background

Neck pain is a common musculoskeletal condition [1] and is the fourth leading cause of years lost to disability [2]. Neck pain along with low back pain is estimated to be the third-largest condition of health care spending, at 87.6 billion US dollars per year [3]. The Global Burden of Diseases (GBD) reported that neck and low back pain spending increased the most relative to any other chronic disease from 1996 through 2013 [4] and the rise in cost did not correlate with improvements in physical function [5].

Neck pain is one of the most common reasons patients seek healthcare [6] and the majority of patients initially seek care from a primary care provider [7]. Primary care providers often recommend medication, imaging, specialist referral or a combination of recommendations [8], but evidence is often lacking for many of these treatment or diagnostic approaches [9]. Recently published clinical practice guidelines for patients experiencing neck pain recommend interventions often provided by physical therapists such as structured patient education, range of motion exercises, spinal mobilization or manipulation with exercise as initial management strategies [10, 11].

Emerging evidence in patients with neck pain and existing evidence in patients with low back pain [12–16] suggest that seeking care from a physical therapist early during an episode of neck pain, including through direct access, is associated with decreased costs and healthcare utilization as well as improved or comparable clinical outcomes [17, 18]. But the effect of timing of physical therapy consultation on healthcare utilization and costs has yet to be widely evaluated in patients with neck pain alone. The improvement in cost savings and decreased healthcare utilization associated with early physical therapy consultation are thought to be attributed to quicker initiation of physical therapy using appropriate interventions [18] and decreasing exposure to unneeded diagnostic testing, interventions and high-risk pain management strategies such as prescribing opioids [19–24]. These findings indicate that physical therapists may be an appropriate front line provider for patients to consult early during an episode of neck pain [25].

Therefore, in this study we wanted to examine the association of the timing of physical therapy consultation in patients seeking care for neck pain with 1-year healthcare utilization and costs. The purpose of this study was to compare 1-year healthcare utilization of imaging, spinal injections, opioid prescription and costs between patients who consulted a physical therapist “early” (within 14 days), “delayed” (within 15–90 days) and “late” (91–364 days) when seeking care for neck pain for which they had not done so in the previous 90 days.

## Methods

### Patients

Patients consulting a physical therapist for a complaint of neck pain from January 1 2012–June 30, 2013 who were continuously insured under one plan, University of Utah Health Plans (UUHP), were eligible to be included in the analysis. Patients insured under UUHP were participating under a Medicaid managed care plan (a government subsidized plan) or a privately insured, employer-based plan. Patients included in this study sought care from hospital-based or an ambulatory physical therapy clinic in Salt Lake City, Utah and surrounding coverage areas. This study was approved by the University of Utah Institutional Review Board.

We identified patients with a new consultation with a healthcare provider for a diagnosis of neck pain using claims data on the basis of the following International Classification of Diseases-Ninth Revision (ICD-9) codes: 721.0, 721.1, 722.0, 722.4, 722.71, 722.81, 722.91, 723.0–723.9, 739.0, 739.1, and 847.0. We defined the date of the first consultation with a healthcare provider with a neck pain ICD-9 code as the index visit. We only included patients with an ICD-9 diagnosis of neck pain on the index visit who did not have a recorded healthcare encounter in the preceding 90 days, in order to reflect a sample of patients seeking care for a new episode of neck pain. The 90-day washout period was used to provide an adequate amount of time to reflect a pain-free state while acknowledging the biases associated with a washout period less than 1 year [26]. Therefore, we excluded any patients who had a neck pain ICD-9 code associated with any claim in the preceding 90 days from the index visit.

### Identifying the timing of physical therapy

We further identified patients who sought care from a physical therapist from billed procedure and revenue codes for physical therapy in the claims data (Additional file 1). To determine the timing of physical therapy consultation, we calculated the number of days between the index visit (first consultation with a healthcare provider) and first physical therapy consultation. We further identified three groups of patients seeking care for a new episode of neck pain. If a patient consulted a physical therapist on the index visit or within 14 days of the index visit, we categorized these patients as receiving “early physical therapy consultation”. If patients consulted a physical therapist within 15–90 days of the index visit, they were categorized as receiving “delayed physical therapy consultation”; if patients consulted a physical therapist after 91 days to any time within the following year from the index visit, they were categorized as receiving “late physical therapy consultation”. The early and delayed physical therapy consultation groups were selected based on previously published literature that described early physical therapy consultation within 14 days and delayed consultation between 14 and 90 days. [27] The late physical therapy consultation group was used as the reference group in analyses.

### Comorbidities

We wished to identify comorbidities that may influence physical therapy outcomes, neck pain prognosis or healthcare seeking behaviors from recorded ICD-9 codes in the claims data within the 1-year period following the index date. We recorded the following provider-entered comorbidities: low back pain [21], fibromyalgia [28], chronic or generalized pain [29], substance abuse, depression and anxiety [30], tobacco use and obesity (see Additional file 1 for ICD-9 codes used for co-morbidity identification).

### Exclusion criteria

**Fig. 1:**
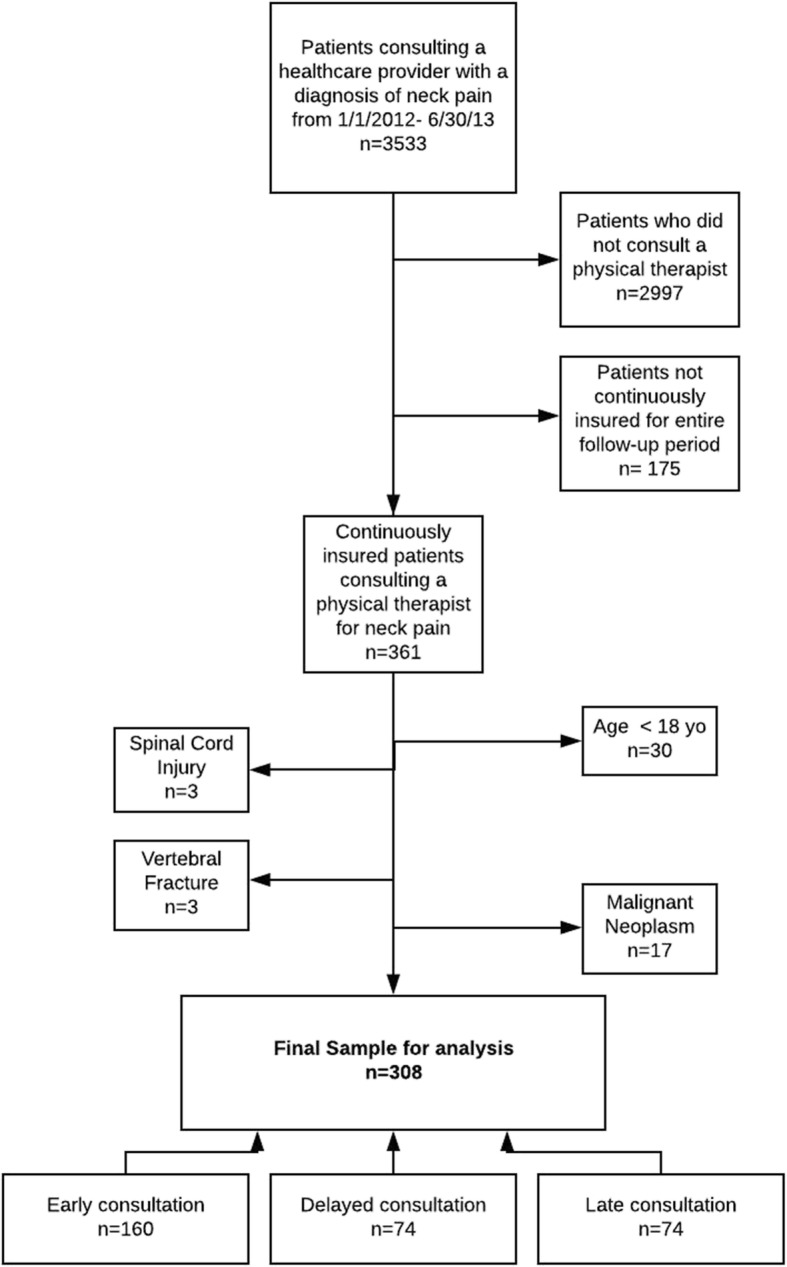
Sample derivation

We excluded patients younger than 18 years of age (*n* = 30) and patients with ICD-9 codes for diagnoses that may adversely affect healthcare costs and utilization such as a diagnosis of a spinal cord injury (n = 3), vertebral fracture (*n* = 3) or malignant neoplasm (*n* = 17). ICD-9 codes for comorbidities were recorded any time with in the 1-year time period from the index visit. See Fig. 1 for sample derivation and Additional file 1 for ICD-9 codes for exclusion.

### Healthcare process variables

We identified process variables associated with the episode of care for neck pain. We recorded the proportion of patients who were privately insured versus insured under Medicaid managed care with UUHP and we recorded the UUHP concurrent risk score, an indicator of health and cost risk [31]. We calculated the healthcare episode of care (HC-EOC) as the number of days from the index visit to the last provider encounter with a recorded ICD-9 code for neck pain. The duration of physical therapy treatment (PT-DOT) was calculated as the number of days from the first physical therapy encounter to the last recorded physical therapy encounter. We also calculated the number of physical therapy visits during the PT-DOT. Median and Interquartile Range (IQR) was reported with HC-EOC, PT-DOT and for number of physical therapy visits.

### Healthcare utilization outcome variables and cost

We wished to explore the association of timing of physical therapy on interventions with conflicting evidence for effectiveness or indication [22–24, 32]. We identified healthcare utilization outcomes from billed procedure codes which has an associated ICD-9 neck pain diagnosis for a 1-year period following the index visit (see Additional file 2). Healthcare utilization outcomes were recorded from the index visit and the following 1-year and may have occurred before and after the physical therapy consultation. We identified patients who received cervical spine injections or nerve blocks; imaging of the cervical spine (MRI, CT and X-ray); or were prescribed an opioid, as identified by therapeutic class codes for Opioids in the claims database (H3A, H3H, H3M, H3N, H3U, H3R), within 1 year following the index visit (Additional file 2). We recorded the billed amounts (costs) from the claims database for all procedures, visits and equipment associated with a neck pain related ICD-9 diagnosis in the 1-year following the index visit.

### Data analysis

STATA 14.2 was used to conduct all statistical analyses. Baseline characteristics, comorbidities, healthcare process variables and unadjusted healthcare utilization variables were compared between physical therapy consultation groups using one-way ANOVAs for continuous variables and chi-squared tests for categorical variables. When comparing the HC-EOC, PT-DOT and number of physical therapy visits, Kruskal-Wallis tests were used due to violations of assumption of normality [33].

Logistic regression was used to compare the odds of healthcare utilization of injections; imaging (MRI, CT or X-ray); and being prescribed an opioid within 1 year from index visit between physical therapy timing groups. Based on previous literature, the covariates of age [34] and gender [34] were included in each model. Based on univariate comparisons, comorbid low back pain [14] and comorbid chronic pain [6] and comorbid substance abuse were also included in each model. Insurance plan type (private versus Medicaid managed care) and UUHP concurrent risk score were also added to the models to account for health-system factors that may affect utilization outcomes. The late physical therapy consultation group was used as the reference group in all analyses and no interaction terms were included in the models.

Mean 1-year neck pain-related billed healthcare costs were compared between physical therapy consultation groups using generalized linear modeling (GLM) with gamma distribution and log link function. GLM was used to allow for parametric analytic methods while accommodating non-normal distribution of cost in order to make inferences about the mean costs directly [35–37]. Both unadjusted and adjusted analyses were performed. Covariates in the adjusted model included age [34], gender [34], comorbid low back pain [14], comorbid chronic pain [6], comorbid substance abuse, UUHP concurrent risk score and insurance plan type (Medicaid or private). No interaction terms were included in the model.

## Results

A total of 3533 patients with a new neck pain-related encounter with a healthcare provider were identified. Of those patients, 15.1% (*n* = 536) had a consultation with a physical therapist within 1-year following the index visit. After patients who were not continuously insured (*n* = 175) and the exclusion criteria were applied, 308 patients remained eligible for analysis. Fifty-two percent of patients (*n* = 160) consulted a physical therapist within 14 days of index visit (“early physical therapy consultation”) and of the patients who consulted a physical therapist within 14 days of index visit, 61% (*n* = 98) consulted a physical therapist on the index visit as the first contact provider for neck pain. Twenty-four percent (*n* = 74) of patients consulted within 15–90 days (“delayed physical therapy consultation”) and 24% (n = 74) of patients consulted a physical therapist between 91 days and 364 days after the index visit (“late physical therapy consultation”). The mean age of patients in the sample was 48.7(14.5) years of age. Patients in the early physical therapy consultation group were the youngest (48 years of age (15.2)) compared to delayed and late physical therapy consultation groups, with patients reporting 49.8(14.0) and 49.0(13.6) years of age respectively (*p* = 0.67). The majority of the sample was female (69.2%) and there was not a significant difference in sex between timing groups (*p* = 0.86). Patients did not differ in the prevalence of comorbid depression (*p* = 0.15), anxiety (*p* = 0.98), fibromyalgia (*p* = 0.59), or obesity (*p* = 0.95). However, groups did differ in the prevalence of low back pain (*p* = 0.01), chronic or generalized pain (*p* < 0.001), substance abuse (*p* = 0.01) and tobacco use (*p* = 0.05) with the late physical therapy consultation group demonstrating the highest prevalence’s of low back pain (81.1%), chronic or generalized pain (47.3%), substance abuse (23.0%) and tobacco use (24.3%). (Table 1).

**Table 1:** Baseline Characteristics and Unadjusted Healthcare Process and Utilization Variables

	Total Sample *N* = 308	Early Physical Therapy Consultation (*n* = 160)	Delayed Physical Therapy Consultation (*n* = 74)	Late Physical Therapy Consultation (n = 74)	*P*
Patient Demographics					
Age	48.7 (14.5)	48.0 (15.2)	49.8 (14.0)	49.0 (13.6)	0.67
Sex (% female)	69.2%	68.8%	71.6%	67.6%	0.86
Comorbidities					
Low Back Pain	66.2%	62.5%	59.5%	81.1%	0.01
Chronic or generalized pain	29.9%	21.3%	31.1%	47.3%	< 0.001
Substance abuse	14.9%	9.4%	18.9%	23.0%	0.01
Depression	34.7%	33.8%	28.4%	43.2%	0.15
Anxiety	26.6%	26.9%	25.7%	27.0%	0.98
Fibromyalgia	21.8%	21.3%	18.9%	25.7%	0.59
Tobacco use	16.2%	11.9%	17.6%	24.3%	0.05
Obesity	15.6%	15.0%	16.2%	16.2%	0.95
Healthcare Process variables					
Plan type (% privately insured)	29.2%	30.6%	25.7%	29.7%	0.74
Duration of Healthcare Episode of Care (HC-EOC) (Days) (median, IQR)	155 (284)	49 (206)	139 (256)	319 (103)	< 0.001
Duration of PT Treatment (PT-DOT) (Days) (median, IQR)	22 (58)	22 (68)	22 (47)	13 (76)	0.5
Number of PT visits (median, IQR)	3 (6)	3 (7)	4 (6)	3 (4)	0.17
Healthcare Utilization Outcomes					
Spinal Injections	35.1%	20.0%	50.0%	52.7%	< 0.001
Prescribed opioids in 1-year from the index visit	62.7%	55.0%	59.5%	82.4%	0.03
Imaging					
MRI (%)	22.7%	10.6%	31.1%	40.5%	< 0.001
X-ray(%)	41.2%	27.5%	55.4%	56.8%	< 0.001
CT	7.1%	3.8%	5.4%	16.2%	< 0.001

### Process outcomes (Table 1)

The percentage of patients who were privately insured in the sample was 29.2% (*n* = 149) and percentages were similar across all groups (*p* = 0.74). The median duration of the HC-EOC for the entire sample was 155 days (IQR 284). The early physical therapy consultation group had the shortest HC-EOC with a median of 49 days (IQR 206) followed by delayed physical therapy consultation group 139 days (IQR 256) and the late physical therapy consultation group 319 days (IQR 103) (*p* < 0.001). The median number of physical therapy visits in the sample was 3 visits (IQR 6) and median PT-DOT was 22 days (IQR 58). Timing groups did not significantly differ in the number of physical therapy visits (*p* = 0.17) or the PT-DOT (*p* = 0.50).

### 1-year healthcare utilization outcomes (Table 2)

#### Spinal injections

The percentage of patients who received spinal injections over a 1-year period in the entire sample was 35.1% (*n* = 108). Over half of patients in the late consultation group received injections 52.7% (*n* = 39) and 50% of the patients in the delayed consultation group received injections (*n* = 37), with only 20.0% (*n* = 32) of patients in the early consultation group received injections. In adjusted analyses, the odds of receiving a spinal injection were increased in the delayed physical therapy consultation group (aOR = 5.34, 95%CI: 2.74–10.41), and in the late physical therapy consultation group (aOR = 4.36, 95%: CI 2.26–8.45) in comparison to the early physical therapy consultation group.

**Table 2:** Adjusted Odds Ratios of Healthcare Utilization

	Early Physical Therapy Consultation (*n* = 160)	Delayed Physical Therapy Consultation (*n* = 74)	Late Physical Therapy Consultation (*n* = 74)
Injections	REF	5.34 (2.74, 10.41)	4.36 (2.26, 8.45)
Prescribed Opioids			
1-year period from the index visit	REF	1.24 (0.69, 2.31)	2.79 (1.35, 5.79)
Imaging			
MRI	REF	4.61 (2.17, 9.78)	4.68 (2.25,9.74)
X-ray	REF	2.73 (1.49, 5.00)	2.97 (1.61, 5.47)
CT	REF	1.51 (0.39, 5.77)	3.36 (1.14, 9.97)

Covariates in models: plan type, UUHP concurrent risk score, age, sex, comorbid LBP, substance abuse and chronic pain

#### Prescription of opioids

Overall, 62.7% (*n* = 193) of patients in the sample were prescribed opioids in the 1-year following the index visit. In univariate comparisons, the highest proportion of patients prescribed opioids were in the late physical therapy consultation group, with 82.4% (*n* = 61), 59.5% (*n* = 44) in the delayed physical therapy consultation group and 55% (*n* = 88) in the early consultation group. After adjusting for covariates, receiving late physical therapy consultation (aOR = 2.79, 95%CI 1.35–5.79) was associated with an increased odds of being prescribed an opioid during the 1-year following the index visit compared to the early physical therapy consultation group, but the delayed physical therapy consultation group did not differ in the odds of being prescribed an opioid (aOR = 1.24, 95%CI 0.69–2.31) compared to the early physical therapy consultation group.

#### Imaging

##### Magnetic resonance imaging (MRI)

The percentage of patients undergoing an MRI in the sample was 22.7% (*n* = 70) in the 1-year following the index visit. The smallest percentage of patients undergoing an MRI was in the early physical therapy consultation group (10.6%, *n* = 17) followed by the delayed physical therapy consultation group (31.1%, *n* = 23) and the late physical therapy consultation group (40.5%, *n* = 30). In adjusted analyses, the odds of undergoing an MRI was increased in both the delayed physical therapy consultation group (aOR = 4.61, 95%CI 2.17–9.78) and the late physical therapy consultation group (aOR = 4.68, 95%CI 2.25–9.74) compared to the early physical therapy consultation group.

##### Radiographs

In the total sample, 41.2% of patients (*n* = 127) underwent a cervical spine X-ray. In unadjusted analyses, the largest percentage of patients who underwent an X-ray were in the late physical therapy consultation group (56.8%, *n* = 42) followed by the delayed physical therapy consultation group (55.4%, *n* = 41) and the early physical therapy consultation group (27.5%, *n* = 44). In adjusted analyses, the delayed physical therapy consultation group (aOR = 2.73, 95%CI 1.49–5.00) and the late physical therapy consultation group (aOR = 2.97, 95%CI 1.61–5.47) demonstrated an increase in the odds of undergoing an X-ray compared to the early physical therapy consultation group.

##### Computed tomography (CT) scan

Only a small percentage of patients in the total sample, 7.1% (*n* = 22), received a CT scan of the cervical spine. By group, the highest percentage of patients undergoing a CT scan were in the late physical therapy consultation group (16.22%, *n* = 12) followed by the delayed physical therapy consultation group (5.4%, *n* = 4) and the early physical therapy consultation group (3.8%, *n* = 6). In adjusted analyses, the odds of receiving a CT were increased (aOR = 3.36, 95%CI 1.14–9.97) in the late physical therapy consultation group compared to the early physical therapy consultation group. The delayed physical therapy consultation group did not differ in the odds of undergoing a CT compared to the early physical therapy consultation group (aOR = 1.51 95%CI 0.39–5.77).

### 1-year healthcare costs (Tables 3 and 4)

The unadjusted mean healthcare cost for the early physical therapy consultation group was $1362, (95%CI $845 - $1879), followed by the delayed physical therapy consultation group $2076, (95%CI $1304 - $2847) and late physical therapy consultation group $6763, (95%CI $3392 - $10135). The relationship of cost in the physical therapy consultation groups remained the same in the adjusted analyses, where patients in the early physical therapy consultation group demonstrated the lowest 1-year healthcare cost, $1853 (95%CI $1172 - $2536), followed closely by the delayed physical therapy consultation group, $2917 (95%CI $1969 - $3866) and the highest 1-year cost was seen in the late physical therapy consultation group $4026, (95%CI $2377 - $5674), (*p* < 0.001). The 1-year healthcare costs in the late physical therapy consultation group were $2172 (95% CI $557 - $3786, *p* < 0.01) higher than the early physical therapy consultation group. There was not a significant mean cost difference between the late physical therapy consultation group ($-1108, 95% CI $-2850 - $634, *p* < 0.21) and the delayed physical therapy consultation group. The 1-year healthcare costs in the delayed physical therapy consultation group were $1063 (95% CI $138 - $1988, *p* < 0.02) higher than the early physical therapy consultation group.

**Table 3:** Mean billed 1-year Healthcare costs

	Early Physical Therapy Consultation (*n* = 160)	Delayed Physical Therapy Consultation (*n* = 74)	Late Physical Therapy Consultation (*n* = 74)
Unadjusted Mean 1-year billed cost in USD (mean, 95%CI)*	$1362 ($845,$1879)	$2076 ($1304, $2847)	$6763 ($3392, $10135)
Adjusted Mean 1-year billed cost in USD (mean, 95%CI)*	$1853 ($1172, $2536)	$2917 ($1969, $3866)	$4026 ($2377, $5674)

Covariates in models: HC-EOC, plan type, UUHP concurrent risk score, age, sex, comorbid LBP, substance abuse and chronic pain

*P < 0.001

**Table 4:** Adjusted Mean Difference in 1-year Billed Cost (US dollars) between Groups

Group Comparisons	Adjusted Mean difference in 1-year billed cost (US dollars)	*P*
Delayed Physical Therapy Consultation Group compared to Early Physical Therapy Consultation Group	$1063 ($138, $1988)	0.02
Late Physical Therapy Consultation Group compared to Early Physical Therapy Consultation Group	$2172 ($557, $3786)	0.01
Late Physical Therapy Consultation Group compared to Delayed Physical Therapy Consultation Group	$1108 ($634, $2850)	0.21

Covariates in models: HC-EOC, plan type, UUHP concurrent risk score, age, sex, comorbid LBP, substance abuse and chronic pain

## Discussion

This study used retrospective claims data to examine the association of the timing of physical therapy consultation on 1-year healthcare utilization and costs in patients seeking care for neck pain. We found that consulting a physical therapist late after seeking care for a new episode of neck pain (greater than 90 days after the index visit) was associated with increased healthcare utilization including spinal injections, imaging (X-ray, MRI and CT scan) and opioid prescription within 1-year following the index visit. Late physical therapy consultation was associated with an average $2172 increase in 1-year billed healthcare costs in comparison to the early physical therapy consultation group. Delaying physical therapy consultation (between 15 and 90 days after an index visit) was associated with similar increases to the late physical therapy consultation group in 1-year healthcare costs ($1063), utilization of spinal injections and imaging(except CT) compared to early physical therapy consultation. Current trends in healthcare spending indicate that healthcare costs are becoming unsustainable for payers and patients and is not resulting in improved outcomes. The findings from our study indicate that consulting a physical therapist early for neck pain, within 14 days of an index visit, may provide an opportunity to mitigate downstream healthcare utilization while containing costs.

The findings from our study in patients with neck pain are consistent with what has been reported in the neck and low back pain literature. In patients consulting a physical therapist delayed or late in our study, we found that there was a similar increase in the risk of being prescribed an opioid, spinal injections or advanced imaging (MRI and CT) in comparison to other published studies [13, 27, 38] but the magnitude of the risk was higher and the estimates were less precise with larger confidence intervals in this study. The greater increase in risk may be reflective of the difference in practice patterns of providers treating neck pain alone verses treating both neck and low back pain or back pain alone, such as in other published studies. In our study, the providers may be more likely to use diagnostic testing or more invasive treatments prior to initiating physical therapy, potentially due to the lack of preponderance of published guidelines for best practices for treating neck pain. Conversely, early physical therapy consultation may shield patients from this utilization pattern. Moreover, the reduction in cost associated with early physical therapy in our study (Adjusted Mean 1-year billed cost in early group: $1853, 95%CI: $1172 - $2536) is very similar to recently published costs in patients seeking care through direct access for neck and low back pain in a different health system (mean cost: $1542, 95%CI: $108 - $2976) [18]. These findings indicate that there is an association with consulting a physical therapist early with 1-year healthcare costs that extend beyond our study. The summary of the findings of this study have preliminarily showed that the timing of physical therapy may be important when considering downstream healthcare utilization and costs for an episode of neck pain.

This study has numerous strengths. This is the first study to examine the relationship between the timing of physical therapy consultation on 1-year healthcare utilization of opioids, imaging and injections as well as costs in patients with neck pain. Moreover, this study provides insights about how patients with neck pain utilize the health care system in relation to consulting with a physical therapist. We provided descriptive data on utilization rates of spinal injections, imaging and opioid use as well as described physical therapy process outcomes between physical therapy timing groups. This study adds to the current literature in patients with neck pain and provides information on how the timing of physical therapy may influence the healthcare experience for patients, filling a critical gap in the literature beyond what is reported in patients with low back pain.

Our study also has limitations. The most notable limitation of the study is the retrospective study design, from a single health care insurer in one geographic location utilizing primarily claims-based data. Therefore, our findings cannot be interpreted as causal or considered widely generalizable based on our study design and source of data. Furthermore, we were unable to measure factors which may have affected outcomes such as patient preference, severity of neck pain, patient demographics such as socioeconomic status or education, specific physical therapy interventions or practice patterns or variations across physical therapy clinics or locations. Although baseline characteristics, process variables and comorbidities were adjusted for in the analyses, there is a potential for the influence of confounding factors that were unaccounted for the in the analyses and a likelihood of selection bias. Lastly, this study examines the effect of timing of physical therapy from a health system perspective; we are unable to determine the relationship healthcare utilization and costs with access to care, patient reported outcomes or patient preference.

## Conclusions

This study has found an association with the timing of physical therapy consultation on healthcare utilization and costs, where delayed and late physical therapy consultation is associated with increased costs and overall healthcare utilization, particularly of healthcare services with conflicting evidence for effectiveness [22–24]. Future studies need to further explore improving earlier access to physical therapy for patients with neck pain. Specifically future studies need to determine the effect of early physical therapy consultation within the primary care setting or through direct access [39] in a formal randomized controlled trial.

## Additional files

Additional file 1: ICD-9 codes used for defining sample, exclusion criteria and defining comorbidities. (DOCX 14 kb)

Additional file 2: ICD-9 codes used for healthcare utilization and opioid use. (DOCX 13 kb)

## Abbreviations

CT: Computed Tomography
HC-EOC: Duration of Healthcare Episode of Care
LBP: Low back pain
MRI: Magnetic Resonance Imaging
PT: Physical therapist
PT-DOT: Duration of PT Treatment
UUHP: University of Utah Health Plans
