# Supplementary material for: Timing of physical therapy consultation on 1-year healthcare utilization and costs in patients seeking care for neck pain: a retrospective cohort
Source: BMC Health Serv Res. 2018 Nov 26;18:887. doi: 10.1186/s12913-018-3699-0 (PMC6258489; doi:10.1186/s12913-018-3699-0)
Supplement: Supplementary file 1 — ICD-9 codes used for defining sample, exclusion criteria and defining comorbidities. (DOCX 14 kb) [file 12913_2018_3699_MOESM1_ESM.docx]

**Additional file 1 ICD-9 codes used for defining sample, exclusion criteria and defining comorbidities**

| **ICD-9 Codes Defining Neck Pain Cohort** | |
| --- | --- |
| Neck Pain Diagnoses | 721.0(cervical spondylosis), 721.1(cervical spondylosis with myelopathy), 722.0 (cervical disc displacement), 722.4(cervical disc degeneration), 722.71(Intervertebral disc disorder with myelopathy, cervical region), 722.81 (postlaminectomy syndrome, cervical region), 722.91 (Other and unspecified disc disorder, cervical region), 723.0 (cervical spine stenosis), 732.1 (cervicalalgia), 723.2 (cervicocranial syndrome)723.3 (cervicobrachial syndrome), 723.4 (brachial neuritis), 723.5 (torticollis NOS), 723.6 (panniculitis of neck), 723.7 (ossification of cervical ligament), 723.8 (cervical syndrome NEC), 723.9 (neck disorder, NOS), 739.0 (nonallopathic lesions, head region), 739.1(nonallopathic lesions, cervical region), 847.0 (sprain of the neck) |
| **Procedure and Revenue Codes Identifying Physical Therapy Utilization** | |
| Procedure code | 97001, 97002, T1015 |
| Revenue Code | 0420, 0421, 0424 |
| **ICD-9 Codes Defining Exclusion Criteria** | |
| Cervical Vertebral Fracture | 733.13  805.01-805.07, 805.10-805.17 |
| Cervical Spinal Cord Injury | 806.00-806.19; 952.0-952.09 |
| Cancer of Cervical Spine(Malignant Neoplasms) | 140.XX-149.XX, 150.XX-159.XX, 160.XX-169.XX, 170.XX-179.XX, 180.XX-189.XX, 190.XX-199.XX, 200.XX-209.0, 209.1-209.3 |
| **ICD-9 Codes Defining Comorbidities** | |
| Chronic or Generalized Pain | 338.21, 338.28, 780.96, 338.29, 338.4, 338.2 |
| Low Back Pain | 739.3, 721.3, 722.1, 722.52, 722.73, 722.83, 722.93, 724.xx, 729.2, 737.3, 756.11, 756.12, 846.xx, 847.2, 847.3, 847.9, 922.31 |
| Substance abuse | 305.XX |
| Depression | 296.XX, 311.XX, 309.XX |
| Anxiety | 300.XX |
| Fibromyalgia | 729.1 |
| Tobacco use disorder | 305.1, V15.82 |
| Obesity | 278.XX, V85.XX |
