# Supplementary material for: Timing of physical therapy consultation on 1-year healthcare utilization and costs in patients seeking care for neck pain: a retrospective cohort
Source: BMC Health Serv Res. 2018 Nov 26;18:887. doi: 10.1186/s12913-018-3699-0 (PMC6258489; doi:10.1186/s12913-018-3699-0)
Supplement: Supplementary file 2 — ICD-9 codes used for healthcare utilization and opioid use. (DOCX 13 kb) [file 12913_2018_3699_MOESM2_ESM.docx]

**Additional file 2 ICD-9 codes used for healthcare utilization and opioid use.**

|  | **Procedure Codes Defining Healthcare Utilization** |
| --- | --- |
| Cervical Spine Surgery | 22551, 22552, 63075, 63076, 63050, 63051, 63045, 63048, 63015, 22554, 22585, 22600, 22614, 63020, 63001, 22595 |
| Anesthetic spinal injections or nerve blocks | 64405, 62310, 64400, 64450, 64479, 64492, 64491, 64490, 64402, 20552, 20553 |
|  | **Therapeutic Classes Defining Opioids** |
| H3A: Long and Short Acting Opioids  H3H: Analgesics Narcotic, Anesthetic Adjunct  H3M: Narcotic Analgesic, Non-salicylate Analgesic, Barbiturate & Xanthine Combination  H3N: Analgesics, Narcotics Agonist &NSAIDs, COX Inhibitor type Combination  H3U: Narcotic Analgesic & Non-salicylate Analgesic Combination; Narcotic & Salicylate Analgesics  H3R: Barbiturate & Xanthine Combination | |
